# Exosome-transferred hsa_circ_0014235 promotes DDP chemoresistance and deteriorates the development of non-small cell lung cancer by mediating the miR-520a-5p/CDK4 pathway

**DOI:** 10.1186/s12935-020-01642-9

**Published:** 2020-11-17

**Authors:** Xueliang Xu, Rong Tao, Liying Sun, Xia Ji

**Affiliations:** 1 Department of Respirtory and Critical Care Medicine, Linyi People’s Hospital, Linyi, Shandong People’s Republic of China; 2 Department of Respirtory and Critical Care Medicine, Intersection of Wuhan Road and Wohu Mountain, Linyi People’s Hospital, Beicheng New District Hospital, Lanshan District, Linyi, Shandong China

**Keywords:** hsa_circ_0014235, Exosome, miR-520a-5p, CDK4, NSCLC

## Abstract

**Background:**

Circular RNAs (circRNAs) play crucial roles in the development and progression of human cancers, including non-small cell lung cancer (NSCLC). However, most of these circRNAs, such as hsa_circ_0014235, are not fully identified in functions and mechanisms.

**Methods:**

The isolated exosomes from serum specimens were identified using transmission electron microscopy (TEM). The expression of hsa_circ_0014235, miR-520a-5p and cyclin-dependent kinase 4 (CDK4) was detected by real-time quantitative polymerase chain reaction (qPCR). For functional assays, cell proliferation, colony formation ability, migration, invasion, cell apoptosis and cell cycle progression were determined using cell counting kit-8 (CCK-8) assay, colony formation assay, wound healing assay, transwell assay and flow cytometry assay, respectively. The expression of CDK4 and other indicated marker proteins was detected by western blot. The predicted target relationship between miR-520a-5p and hsa_circ_0014235 or cyclin-dependent kinase 4 (CDK4) was verified by dual-luciferase reporter assay or RNA immunoprecipitation (RIP) assay.

**Results:**

The expression of hsa_circ_0014235 was notably elevated in NSCLC serum-derived exosomes, tumor tissues and cells. NSCLC serum-derived exosomes promoted NSCLC cell resistance to cisplatin (DDP), cell proliferation, migration and invasion in vitro, as well as tumor growth and DDP resistance in vivo. Hsa_circ_0014235 overexpression enhanced DDP resistance and facilitated cell malignant behaviors. MiR-520a-5p was a target of hsa_circ_0014235, and rescue experiments showed that miR-520a-5p restoration reversed the effects of hsa_circ_0014235 overexpression. Moreover, CDK4 was a target of miR-520a-5p, and rescue experiments showed that CDK4 knockdown reversed the aggressive effects of miR-520a-5p inhibition on NSCLC progression.

**Conclusions:**

Exosome-transmitted hsa_circ_0014235 promoted NSCLC malignant development by mediating the miR-520a-5p/CDK4 regulatory axis.

## Background

Lung cancer is the most common cancer around the world, with high incidence and mortality [1]. Lung cancer is divided into two subtypes, including non-small cell lung cancer (NSCLC; accounting for 85% of all lung cancers) and small cell lung cancer (accounting for 15% of all lung cancers) [2]. Recently, the treatment outcomes and the quality of NSCLC patients’ life have improved a lot. However, the lack of diagnostic techniques and effective biomarkers results in the diagnosis at an advanced stage, and the inevitable drug resistance is developed during chemotherapy [3–5]. These are major obstacles to treatment failure. Therefore, understanding drug resistance mechanisms and identifying underlying biomarkers are essential to improve therapeutic outcomes.

Exosomes are membrane-derived nano-vesicles and considered to be a means of intercellular communication [6]. Exosomes can be detected in various body fluids, such as blood, urine, saliva and ascites, and increasing evidence suggests that exosomes play a vital role in multiple biological processes [7, 8]. Thus, exosomes are considered to be feasible diagnostic biomarkers in the detection of multiple diseases, including cancer [9]. Several studies addressed that exosomes could transfer diverse components out of cells, such as lipids, proteins and non-coding RNAs, which was involved in a series of physiological functions, even the pathogenesis of some diseases [10]. For example, exosomal long non-coding RNAs (lncRNAs), microRNAs (miRNAs) and circular RNAs (circRNAs) from cancer patients have been proposed as novel biomarkers in multiple cancers [11–13].

CircRNAs are a class of structurally stable and widely distributed non-coding RNA molecules, characterized by closed-loop structure [14]. Accumulating studies have demonstrated that circRNAs with regulatory functions are involved in the development of cancers, affecting cell proliferation, apoptosis, migration and invasion [15–17]. Previous study held the view that circRNAs were abundantly and stably existed in exosomes, and serum-derived exosomal circRNAs might distinguish cancer patients from healthy controls [18]. Exosomal circRNAs were thus defined as more promising biomarkers for cancer diagnosis and treatment [18, 19]. Hsa_circ_0014235 is derived from S100 calcium binding protein A2 (S100A2), which is shown in circbank database (http://www.circbank.cn/search.html?selectValue=hsa_circ_0014235). Whether hsa_circ_0014235 plays function in human cancers remains unknown till now.

The circRNA/miRNA/mRNA regulatory network was constructed to address the functional mechanism of circRNA in multiple biological processes in numerous studies [20, 21]. Given that circRNA harbors miRNA response elements (MREs), and circRNA may function as a miRNA sponge to suppress miRNA expression [22]. Besides, miRNA regulates gene expression by binding to 3′ untranslated region (3′UTR) of mRNAs [23]. With the advance of bioinformatics, miR-520a-5p is predicted as a target of hsa_circ_0014235, and cyclin-dependent kinase 4 (CDK4) is predicted as a target of miR-520a-5p. Whether miR-520a-5p and CDK4 are implicated in the hsa_circ_0014235 regulatory pathway needs further exploring.

Herein, we examined the expression of hsa_circ_0014235 in NSCLC serum-derived serum, tumor tissues and cells. In function, we investigated the role of hsa_circ_0014235 on cisplatin (DDP) chemoresistance, cell proliferation, cycle, invasion, migration and apoptosis. Besides, the hsa_circ_0014235/miR-520a-5p/CDK4 axis was assembled to explain the mechanism of hsa_circ_0014235 action in NSCLC. This study aimed to provide evidence for hsa_circ_0014235 as a promising biomarker for NSCLC.

## Materials and methods

### Tissue and serum specimens

This study was implemented with the authorization of the Ethics Committee of Linyi People’s Hospital. NSCLC patients and healthy volunteers were all recruited from Linyi People’s Hospital. Tumor tissues (n = 35) and adjacent normal tissues (n = 35) were collected from NSCLC patients, and serum specimens were collected from NSCLC patients and healthy volunteers by centrifugation. The written informed consent was obtained from each subject before sample collection. All specimens were placed in liquid nitrogen and stored at − 80 ℃ conditions.

### Exosome isolation and transmission electron microscopy (TEM)

Exosomes were isolated from serum specimens using the Exoquick exosome precipitation solution (System BioScience; Palo Alto, CA, USA), the following procedures were in accordant with the protocol.

The morphology of isolated exosomes was investigated by TEM. Briefly, a total of 10 µL exosome pellets were transferred on formvar carbon-coated electron microscopy grids (200-mesh copper), maintaining for 5 min at room temperature, followed by the incubation of 1% uranyl acetate staining for 1 min at room temperature. The grids were washed with phosphate buffered saline (PBS) and placed at room temperature for drying. The morphology of exosomes was observed under a TEM (Hitachi H7500, Tokyo, Japan).

### Western blot

Total protein was extracted by using RIPA lysis (Solarbio, Beijing, China) and quantified by BCA assay kit (Solarbio). Then, the protein was transferred into PVDF membranes (Bio-Rad; Hercules, CA, USA) after separating and then incubated with the 5% skim milk for blocking. The membranes containing the isolated proteins were probed with the primary antibodies (Abcam; Cambridge, MA, USA), including anti-CD63 (ab134045), anti-CD81 (ab109201), anti-matrix metallopeptidase 9 (anti-MMP9; ab76003), anti-Bax (ab32503), anti-E-cadherin (ab40772), anti-Vimentin (ab45939), anti-multidrug resistance-associated protein 1 (anti-MRP-1; ab230948) and anti-CDK4 (ab108357). After culturing at 4 ℃ overnight, the membranes were probed with the HRP-conjugated secondary antibodies (ab150077 and ab205718) at 37 ℃ for 1.5 h. The signals of indicated proteins were visualized by the ECL Western Blotting Substrate (Solarbio) and quantified using Image J software (NIH, Bethesda, MA, USA).

### Real-time quantitative polymerase chain reaction (qPCR)

Total RNA was isolated using the Total RNA Extraction Kit (Solarbio). The integrality and quality of RNA were examined using gel electrophoresis and Nanodrop 2000 (Thermo Fisher Scientific; Waltham, MA, USA). Next, cDNA was synthesized from total RNA using the Universal RT-PCR Kit (M-MLV; Sorlarbio), and the following qPCR assay was conducted using the 2 × SYBR Green PCR Mastermix (Sorlarbio). The relative expression was calculated using the 2^−ΔΔCT^ method, with glyceraldehyde-3-phosphate dehydrogenase (GAPDH) or U6 as the internal reference. The primer sequences were listed as follows: hsa_circ_0014235, F: 5′-GCGACAAGTTCAAGCTGAGT-3′ and R: 5′-GGAAGGTAGTGACCAGCACAG-3′, miR-520a-5p, F: 5′- ACACTCCAGCTGGGCTCCAGAGGG-3′ and R: 5′-CTCAACTGGTGTCGTGGAGTCGGCAATTCAGTTGAGAGTTTGTAC-3′, CDK4, F: 5′-atgttgtccggctgatgga-3′ and R: 5′-caccagggttaccttgatctcc-3′. GAPDH, F: 5′-acccagaagactgtggatgg-3′ and R: 5′-ttctagacggcaggtcaggt-3′, U6, F: 5′-GCTTCGGCAGCACATATACTAAAAT-3′ and R: 5′- CGCTTCACGAATTTGCGTGTCAT-3′.

## Cell lines

NSCLC cells (A549 and H1299) and human bronchial epithelial cells (16HBE; non-cancer cells) were purchased from Bena culture collection (Beijing, China). A549 and H1299 cells were cultured in DMEM (Bena culture collection) containing 10% fetal bovine serum (FBS), and 16HBE cells were cultured in RPMI1640 (Bena culture collection) containing 10% FBS. All cells were cultured in a 37 ℃ incubator supplemented with 5% CO_2_.

### DDP treatment and exosome incubation

A549 and H1299 cells were treated with DDP (Sigma-Aldrich; St. Louis, MO, USA) at different concentrations (0, 1, 2, 4, 8, 16, 32 and 64 µg/mL) for 72 h for cell viability detection. A549 and H1299 cells treated with 5 µg/mL DDP were used in the following experiments. DDP-treated cells in the Exo group were administered with 10 µg/mL exosomes derived from NSCLC serum, and DDP-treated cells in the PBS group were administered with 10 µg/mL exosome-free PBS.

### Cell transfection

Hsa_circ_0014235 overexpression vector (oe-circ) and empty pCD-CIR vector (Vector; control) were provided by Geneseed (Guangzhou, China). MiR-520a-5p mimic and mimic negative control (mimic NC), as well as miR-520a-5p inhibitor and inhibitor negative control (inhibitor NC) were purchased from Ribobio (Guangzhou, China). Small interference RNA targeting CDK4 (si-CDK4) and its negative control (si-NC) were assembled by Ribobio. These oligonucleotides and plasmids were transfected into A549 and H1299 cells alone or together using the Lipofectamine 3000 Reagent (Invitrogen, Carlsbad, CA, USA).

### Cell counting kit-8 (CCK-8) assay

A549 and H1299 cells in 96-well plates treated with DDP at different concentrations for 72 h were then exposed to 10 µL CCK-8 solution (Solarbio) for another 2 h, and cell viability was detected under a microplate reader (Thermo Fisher Scientific) at 450 nm.

For cell proliferation analysis, cells in 96-well plates treated with the indicated concentration of DDP were cultured for 0, 24, 48 or 72 h. Then, 10 µL CCK-8 solution was added into each well, incubating for another 2 h. The absorbance at 450 nm was detected under a microplate reader.

### Colony formation assay

A549 and H1299 cells were seeded into 6-well plates at a density of 200 cells/well. The plates were placed in a 37 ℃ incubator containing 5% CO_2_ for 2 weeks to allow colony growth. The colonies were washed by PBS, fixed with formaldehyde (Beyotime, Shanghai, China) and stained with crystal violet (Beyotime). The number of colonies was counted under a microscope (Nikon, Tokyo, Japan), and the images were recorded.

### Flow cytometry assay

A549 and H1299 cells were treated with DDP to induce apoptosis. For apoptosis assay, cells were collected and resuspended in PBS (6 × 10^4^ cells), following by the sequential addition of 195 µL Annexin V-FITC binding buffer, 5 µL Annexin V-FITC and 10 µL propidium iodide (PI) from the Annexin V-FITC Apoptosis Detection Kit (Beyotime). The treated cells were incubated at room temperature in the dark for 20 min. Flow cytometry assay was performed using a FACScan flow cytometer (BD Bioscience; San Jose, CA, USA).

Cell cycle progression was monitored using the Cell Cycle Analysis Kit (Beyotime). Cells were collected, treated with trypsin and then resuspended in cooled PBS. Next, cells were fixed with 70% ethanol at 4 ℃ overnight. On the second day, ethanol was removed, and cells were washed with PBS and then stained with PI (containing RNase A) at 37 ℃ for 30 min in the dark. The cell distribution was analyzed by a FACScan flow cytometer.

### Wound healing assay

To monitor cell migration, cells were seeded into 6-well plates (3 × 10^4^ per well) until the cells were 90% confluent. A 200 µL pipette tip was utilized to create an artificial wound in a monolayer of cells. Images were captured at specific time points (0 h and 24 h) from at least five fields to ensure the wound closure. Cells were analyzed and photographed under phase-contrast microscopy (Nikon).

### Transwell assay

For invasion assay, 24-well transwell chambers (Corning Incorporated; Corning, NY, USA) with 8.0 micron pore size polyester were coated with Matrigel (Corning Incorporated). A549 and H1299 cells (2 × 10^4^) in serum-removed medium were seeded into the upper wells, and the lower wells were supplemented with fresh culture medium containing 10% FBS. After 24 h incubation at 37 ℃, cells invaded to the lower chamber during incubation were fixed with formaldehyde and stained with crystal violet (Beyotime). The number of cells that invaded to the bottom of chambers was counted using a phase-contrast microscopy (Nikon) in at least five fields.

### Animal experiment

The animal experimental projects were approved by the Animal Care and Use of Committee of Linyi People’s Hospital. BALB/c nude mice (male, 6-week-old, 18–20 g, n = 24) were obtained from Nanjing Junke Bio (Nanjing, China) and maintained in pathogen-free conditions. The mice were divided into 4 groups (n = 6 per group), including PBS group (injection with PBS only), Exo group (injection with NSCLC exosomes only), PBS + DDP (injection with PBS and administration with DDP) and Exo + DDP group (injection with NSCLC exosomes and administration with DDP). In detail, all mice were subcutaneously inoculated with A549 cells (2 × 10^6^) to allow tumor growth. When tumor volume (length × width^2^ × 0.5) reached at 100 mm^3^, mice were injected with PBS or exosomes (30 µg/mice) in tumor nodes every 3 days, lasting for 3 times. Meanwhile, mice in the PBS + DDP or Exo + DDP group were administrated with DDP (2.5 mg/kg) in by intraperitoneal injection every 3 days. During tumor growth, tumor volume was measured once a week, and tumor weight was measured after 5 weeks when all tumor tissues were removed. Tumor tissues were collected for the following analysis.

### Dual-luciferase reporter assay

The binding site between miR-520a-5p and hsa_circ_0014235 or CDK4 was predicted by the online bioinformatics tool Starbase3.0 (http://starbase.sysu.edu.cn/). According to the wild-type binding site between them, the mutant sequence of hsa_circ_0014235 or CDK4 3′UTR fragment was designed, which was mutated at the miR-520a-5p binding site. Luciferase reporter plasmids, including PGL4-hsa_circ_0014235 wt (hsa_circ_0014235 wt), PGL4-hsa_circ_0014235 mut (hsa_circ_0014235 mut), PGL4-CDK4 3′UTR wt (CDK4 3′UTR wt) and PGL4-CDK4 3′UTR mut (CDK4 3′UTR mut), were constructed for dual-luciferase reporter assay to verify the predicted target relationship. 293T cells (Bena culture collection) were cotransfected with miR-520a-5p mimic or mimic NC and one of the above mentioned reporter plasmids. At 48 h post-transfection, the luciferase activity was detected using the dual-luciferase reporter assay (Promega, Madison, WI, USA) according to the protocol.

### RNA immunoprecipitation (RIP) assay

RIP assay was conducted also to validate the predicted target relationship using the EZ-Magna RIP kit (Millipore, Billerica, MA, USA). A549 and H1299 cells were harvested and exposed to RIP lysis, and then the cell lysates were incubated with bead pellets conjugated with antibodies against Argonaute 2 (Ago2; Millipore) or Immunoglobulin G (IgG; Millipore). After washing off unbound materials, the immunoprecipitated RNAs were isolated for qPCR detection.

### Statistical analysis

GraphPad Prism 7.0 software (GraphPad Prism, La Jolla, CA, USA) was used to process the experimental data. All experiments were performed at least in triplicate and the data were displayed as mean ± standard deviation (SD). Student’s *t*-test was used to compare the difference of two groups, and one-way analysis of variance (ANOVA) was used to compare the difference of multiple groups. The correlation of two variables was conducted using the Pearson correlation coefficient. *P* < 0.05 was considered to be statistically significant.

## Result

### Hsa_circ_0014235 was overexpressed in serum-derived exosomes, tissues and cells of NSCLC

**Fig. 1:**
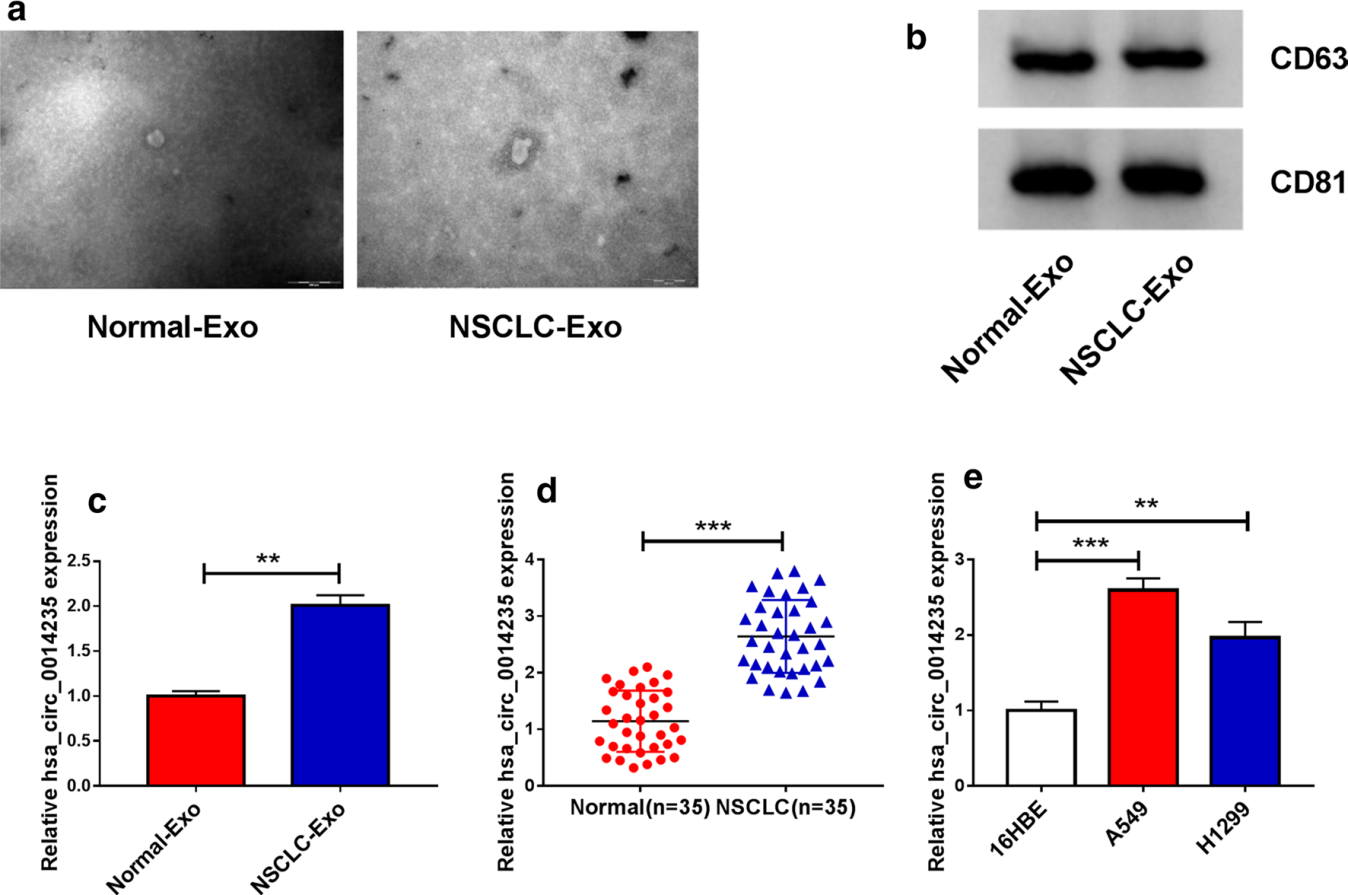
Hsa_circ_0014235 was highly expressed in NSCLC serum-derived exosomes, tumor tissues and cell lines. **a** TEM displayed the morphology of isolated exosomes. **b** The expression of exosome-related markers (CD63 and CD81) was measured by western blot. The expression of hsa_circ_0014235 in exosomes from **c** NSCLC serum or healthy serum, **d** cancer tissues or adjacent normal tissues, and (E) cancer cells (A549 and H1299) or non-cancer cells (16HBE) was detected by qPCR. **P < 0.01, ***P < 0.001

To figure out the expression pattern of hsa_circ_0014235 in NSCLC, we isolated exosomes from serum specimens of NSCLC patients. The image from TEM showed a representative typical lipid bilayer membrane-encapsulated nanoparticles, which represented a morphology of exosomes (Fig. 1a). Several exosome-related markers, including CD63 and CD81, were significantly expressed in serum-derived exosomes (Fig. 1b). The results from qPCR showed that the expression of hsa_circ_0014235 was remarkably increased in serum-derived exosomes from NSCLC patients compared with that from healthy subjects (Fig. 1c). Besides, the expression of hsa_circ_0014235 was also elevated in NSCLC tumor tissues compared with that in adjacent normal tissues (Fig. 1d). Moreover, hsa_circ_0014235 was highly expressed in A549 and H1299 cells compared with that in 16HBE cells (Fig. 1e). We deduced that high expressed of hsa_circ_0014235 in NSCLC might be associated with NSCLC development.

#### NSCLC serum exosomes increased DDP chemoresistance and promoted NSCLC cell proliferation, migration and invasion

**Fig. 2:**
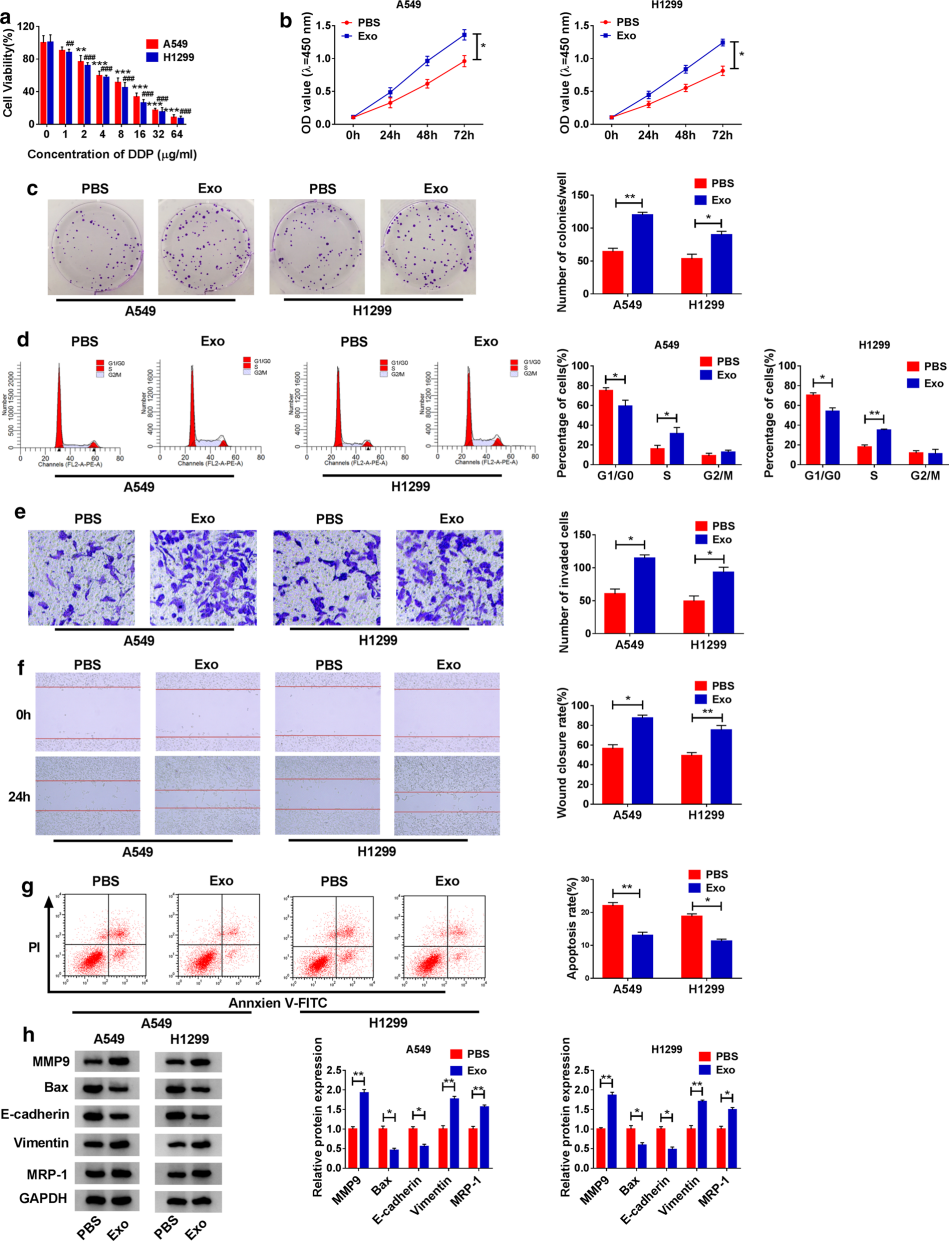
NSCLC serum-derived exosomes induced NSCLC cell malignant behaviors. **a** Cell viability in A549 and H1299 cells treated with different concentrations of DDP was detected by CCK-8 assay. Then, DDP-treated A549 and H1299 cells were incubated with Exo or PBS. In these cells, **b** cell proliferation was monitored in DDP-treated A549 and H1299 cells incubated with Exo or PBS using CCK-8 assay. **c** The ability of colony formation was monitored using colony formation assay. **d** Cell cycle progression was assessed by flow cytometry assay. **e** Cell invasion was investigated by transwell assay. **f** Cell migration was investigated by wound healing assay. **g** Cell apoptosis was detected by flow cytometry assay. **h** The expression of MMP9, Bax, E-cadherin, Vimentin and MRP-1 was measured by western blot. *P < 0.05, **P < 0.01

A549 and H1299 cells were treated with different concentrations of DDP for 72 h, and DDP weakened cell viability in a dose-dependent manner (Fig. 2a). A549 and H1299 cells treated with 5 µg/mL DDP were incubated with NSCLC serum-derived exosomes or PBS and used for the following assays. The ability of cell proliferation was significantly promoted in the exosome group compared to the PBS group (Fig. 2b), and the ability of colony formation was also enhanced in the exosome group compared to the PBS group (Fig. 2c). Besides, the addition of exosomes alleviated cell cycle arrest of A549 and H1299 cells, promoting cell cycle progression (Fig. 2d). Moreover, NSCLC serum-derived exosomes strikingly increased the number of invaded cells and wound closure rate, suggesting that serum exosomes promoted cell migration and invasion (Fig. 2e, f). As expected, NSCLC serum-derived exosomes blocked A549 and H1299 cell apoptosis (Fig. 2g). Subsequently, we quantified the expression of proliferation, apoptosis, migration/invasion and multi-drug resistance-associated proteins in DDP-treated A549 and H1299 cells incubated with NSCLC serum-derived exosomes or PBS. The expression of MMP9, Vimentin and MRP-1 was notably increased, while the expression of Bax and E-cadherin was notably decreased in the exosomes group compared to the PBS group (Fig. 2h). Taken together, NSCLC serum-derived exosomes deteriorated NSCLC malignant behaviors.

#### **NSCLC serum exosomes promoted DDP chemoresistance*in vivo***

**Fig. 3:**
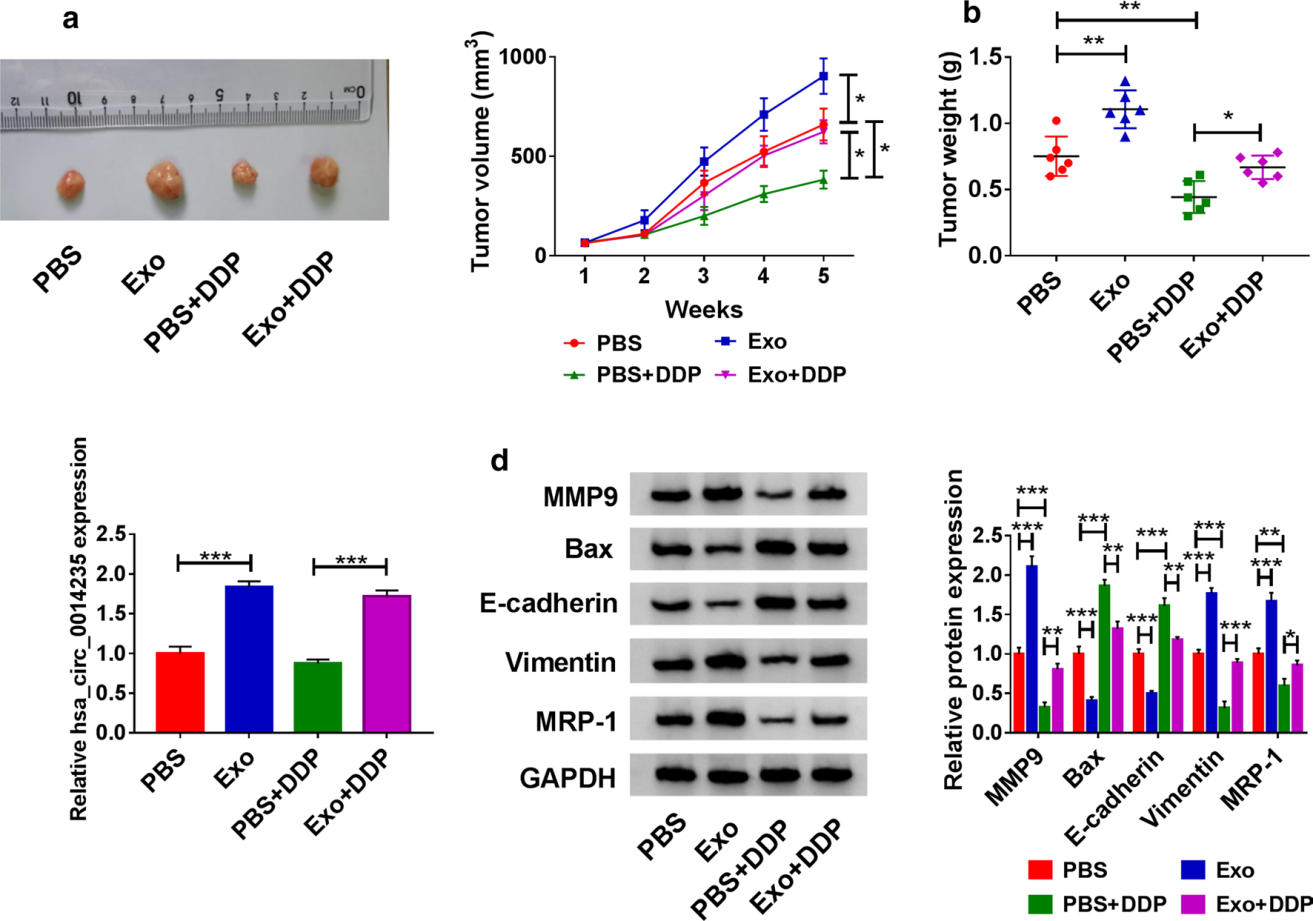
NSCLC serum-derived exosomes strengthened DDP chemoresistance in vivo. A549 cells were injected into the nude mice, and the mice were treated with PBS or Exo and administered with or without DDP. **a** Tumor volume in each group was measured once a week. **b** Tumor weight in each mouse was measured after 5 weeks. **c** The expression of hsa_circ_0014235 in removed tumor tissues was detected by qPCR. **d** The expression of MMP9, Bax, E-cadherin, Vimentin and MRP-1 in removed tumor tissues was measured by western blot. *P < 0.05, **P < 0.01, ***P < 0.001

A549 cells were subcutaneously injected into nude mice. We found that the mice with A549 cells injection treated with NSCLC exosomes showed large tumor volume and tumor weight compared with PBS treatment, and in DDP-administrated mice, NSCLC exosomes treatment weakened the effects of DDP relative to PBS treatment, leading to increased tumor volume and tumor weight (Fig. 3a, b). Finally, we examined the expression of hsa_circ_0014235 in these removed tumor tissues. The data showed that the expression of hsa_circ_0014235 was higher in the Exo group compared with that in the PBS group, and the expression of hsa_circ_0014235 was higher in the Exo + DDP group compared with that in the PBS + DDP group (Fig. 3c). Furthermore, the expression of MMP9, Vimentin and MRP-1 was enhanced, while the expression of Bax and E-cadherin was impaired in the Exo group compared with that in the PBS group (Fig. 3d). Similarly, the expression of MMP9, Vimentin and MRP-1 was enhanced, while the expression of Bax and E-cadherin was impaired in the Exo + DDP group compared with that in the PBS + DDP group (Fig. 3d). All data suggested that NSCLC serum-derived exosomes contributed to DDP chemoresistance *in vivo*.

#### Hsa_circ_0014235 overexpression promoted DDP chemoresistance, leading to increased cell proliferation, migration and invasion

**Fig. 4:**
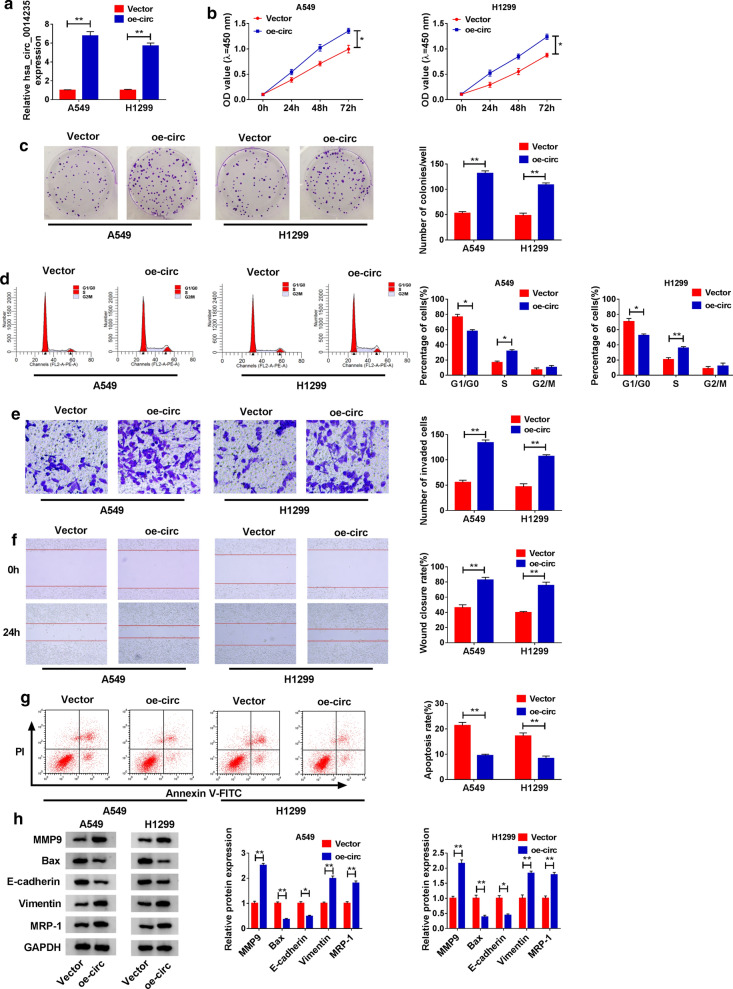
Hsa_circ_0014235 overexpression enhanced DDP resistance and promoted cell malignant behaviors. **a** The efficiency of hsa_circ_0014235 overexpression was checked using qPCR. Then, DDP-treated A549 and H1299 cells were transfected with oe-circ or Vector, and **b** cell proliferation, **c** colony formation ability, **d** cell cycle progression, **e** cell invasion, **f** cell migration and **g** cell apoptosis were examined using CCK-8 assay, colony formation assay, flow cytometry assay, transwell assay, wound healing assay and flow cytometry assay, respectively. **h** The expression of MMP9, Bax, E-cadherin, Vimentin and MRP-1 in these cells was measured by western blot. *P < 0.05, **P < 0.01

The efficiency of hsa_circ_0014235 overexpression was checked, and the expression of hsa_circ_0014235 was notably enhanced in A549 and H1299 cells transfected with oe-circ compared to Vector (Fig. 4a). Then, A549 and H1299 cells transfected with oe-circ or Vector were treated with 5 µg/mL DDP. The transfection of oe-circ significantly reinforced the ability of cell proliferation relative to Vector transfection in A549 and H1299 cells (Fig. 4b). The ability of colony formation was also promoted of A549 and H1299 cells transfected with oe-circ (Fig. 4c). In addition, hsa_circ_0014235 overexpression accelerated A549 and H1299 cell cycle progression (Fig. 4d). Hsa_circ_0014235 overexpression promoted A549 and H1299 cell migration and invasion (Fig. 4e, f), while hsa_circ_0014235 overexpression blocked cancer cell apoptosis (Fig. 4g). Additionally, the expression of MMP9, Vimentin and MRP-1 was heightened, while the expression of Bax and E-cadherin was lessened in A549 and H1299 cells transfected with oe-circ (Fig. 4h). These data manifested that hsa_circ_0014235 overexpression increased the resistance of A549 and H1299 cells to DDP, thus inducing cell malignant behaviors.

### Hsa_circ_0014235 promoted NSCLC development by targeting miR-520a-5p

**Fig. 5:**
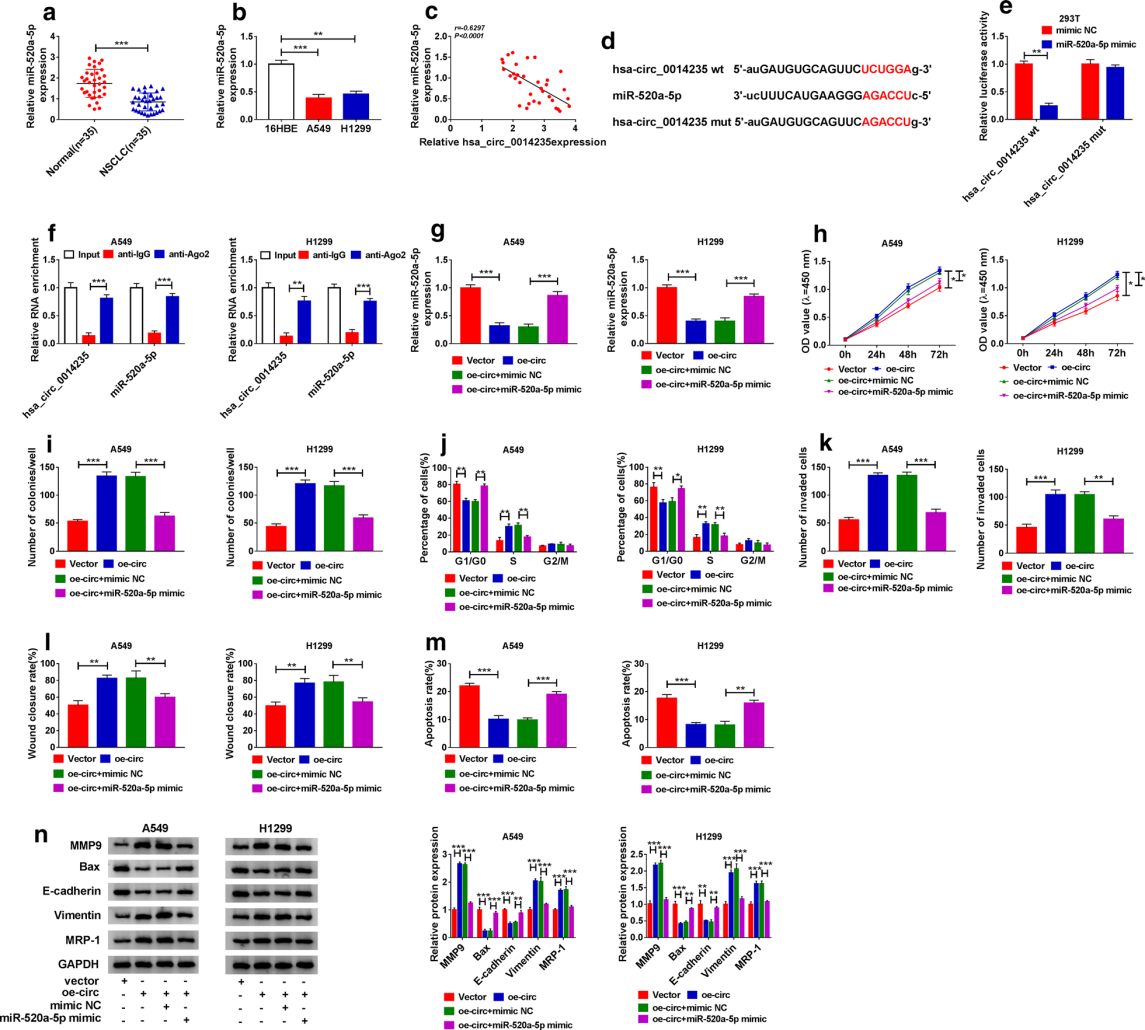
Hsa_circ_0014235 overexpression enhanced DDP resistance and promoted cell malignant behaviors by targeting miR-550a-5p. **a** The expression of miR-550a-5p in cancer tissues and normal tissues was detected by qPCR. **b** The expression of miR-550a-5p in 16HBE, A549 and H1299 cells was detected by qPCR. **c** The correlation between miR-520a-5p expression and hsa_circ_0014235 expression in NSCLC tissues was analyzed by Spearman’s analysis. **d**, **e** Luciferase reporter plasmids were constructed, and dual-luciferase reporter assay was performed to verify the relationship between miR-520a-5p and hsa_circ_0014235. **f** The relationship between miR-520a-5p and hsa_circ_0014235 was also verified by RIP assay. **g** The expression of miR-520a-5p in A549 and H1299 cells transfected with oe-circ, Vector, oe-circ + miR-520a-5p mimic or oe-circ + mimic NC was detected by qPCR. **h** Cell proliferation, **i** colony formation ability, **j** cell cycle progression, **k** cell invasion, **l** cell migration and **m** cell apoptosis in these transfected cells were examined using CCK-8 assay, colony formation assay, flow cytometry assay, transwell assay, wound healing assay and flow cytometry assay, respectively. (N) The expression of MMP9, Bax, E-cadherin, Vimentin and MRP-1 in these cells was measured by western blot. *P < 0.05, **P < 0.01, ***P < 0.001

For mechanism exploration, we identified the target miRNAs of hsa_circ_0014235 and noticed miR-520a-5p that was downregulated in NSCLC tissues and cells relative to normal tissues and non-cancer cells, respectively (Fig. 5a, b). Spearman’s correlation analysis showed that miR-520a-5p expression in NSCLC tissues was negatively correlated with hsa_circ_0014235 expression (Fig. 5c). Besides, according to the wild-type binding site between hsa_circ_0014235 and miR-520a-5p, we mutated the sequence fragment of hsa_circ_0014235 at the binding site with miR-520a-5p and performed dual-luciferase reporter assay (Fig. 5d). The results showed that the cotransfection of miR-520a-5p mimic and hsa_circ_0014235 wt remarkably lessened the luciferase activity in 293T cells relative to the cotransfection of miR-520a-5p mimic and hsa_circ_0014235 mut (Fig. 5e). Moreover, both miR-520a-5p and hsa_circ_0014235 could be abundantly detected in the anti-Ago2 group in RIP assay compared with that in the anti-IgG group (Fig. 5f). Then, we performed rescue experiments to determine whether hsa_circ_0014235 regulated NSCLC development by targeting miR-520a-5p. Firstly, we observed that the expression of miR-520a-5p was notably weakened in A549 and H1299 cells transfected with oe-circ compared to Vector, while miR-520a-5p expression was recovered in cells transfected with oe-circ + miR-520a-5p mimic compared to oe-circ + mimic NC (Fig. 5g). Cell proliferation was promoted in A549 and H1299 cells transfected with oe-circ but impaired in cells transfected with oe-circ + miR-520a-5p mimic (Fig. 5h). Similarly, the ability of colony formation was strengthened by oe-circ transfection but weakened by oe-circ + miR-520a-5p mimic cotransfection (Fig. 5i). The oe-circ transfection alone significantly promoted cell cycle progression, while the cotransfection of oe-circ + miR-520a-5p mimic induced cell cycle arrest (Fig. 5j). Cell invasion and cell migration were induced in A549 and H1299 cells transfected with oe-circ but largely repressed in cells transfected with oe-circ + miR-520a-5p mimic (Fig. 5k, l). Cell apoptosis was notably repressed by hsa_circ_0014235 overexpression but largely stimulated by the reintroduction of miR-520a-5p mimic (Fig. 5m). Additionally, the expression of MMP9, Vimentin and MRP-1 was promoted in A549 and H1299 cells transfected with oe-circ but lessened in cells transfected with oe-circ + miR-520a-5p mimic, while the expression of Bax and E-cadherin was opposite to the expression of MMP9, Vimentin and MRP-1 in these cells (Fig. 5n). These data manifested that hsa_circ_0014235 promoted NSCLC development by targeting miR-520a-5p.

### CDK4, upregulated in NSCLC, was a target of miR-520a-5p

**Fig. 6:**
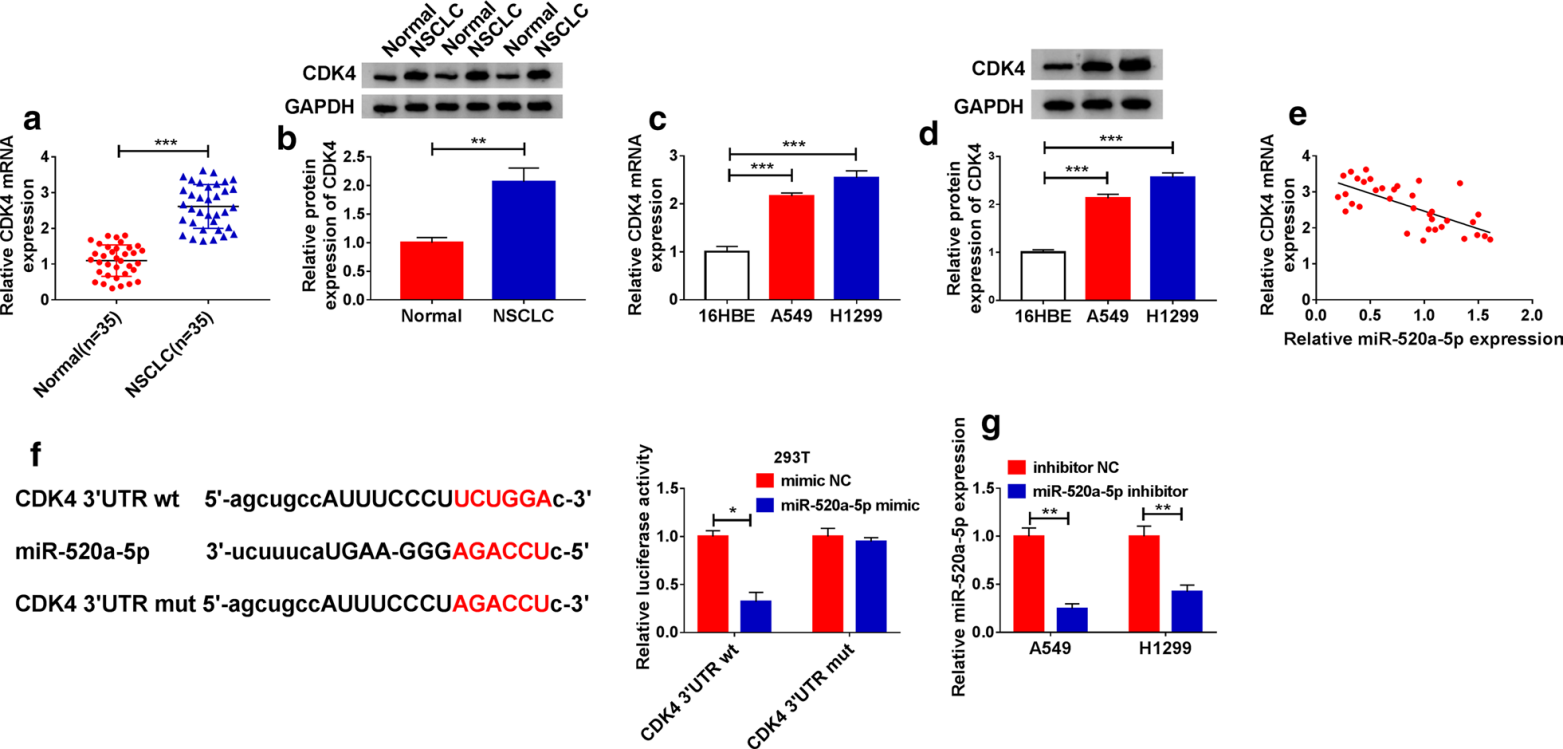
CDK4 was a target of miR-520a-5p. **a**, **b** The expression of CDK4 at mRNA and proteins in NSCLC tissues and normal tissues was detected by qPCR and western blot. **c**, **d** The expression of CDK4 at mRNA and proteins in cancer cells and non-cancer cells was detected by qPCR and western blot. **e** The correlation between CDK4 expression at the mRNA level and miR-520a-5p expression was analyzed by Spearman’s correlation analysis. **f** Luciferase reporter plasmids were constructed, and dual-luciferase reporter assay was conducted to validate the relationship between CDK4 and miR-520a-5p. **g** The efficiency of miR-520a-5p inhibitor was examined by qPCR. *P < 0.05, **P < 0.01, ***P < 0.001

Bioinformatics analysis predicted that CDK4 was a potential target of miR-520a-5p. Further analyses showed that CDK4 expression was elevated in NSCLC tissues compared with that in normal tissues (Fig. 6a, b). Besides, the expression of CDK4 was also enhanced in A549 and H1299 cells compared with that in 16HBE cells (Fig. 6c, d). Spearman’s analysis presented that CDK4 expression at the mRNA level was negatively correlated with miR-520a-5p expression in NSCLC tissues (Fig. 6e). Based on the predicted binding site between miR-520a-5p and CDK4 3’UTR, we constructed the mutant sequence fragment of CDK4 3’UTR to conduct dual-luciferase reporter assay, and the data displayed that the luciferase activity was strikingly lessened in 293T cells transfected with miR-520a-5p mimic and CDK4 3’UTR wt (Fig. 6f), indicating that CDK4 was a target of miR-520a-5p. The inhibitor of miR-520a-5p was then used in the following experiments, the data from qPCR showed that the efficiency of miR-520a-5p inhibitor was available because miR-520a-5p expression was notably decreased in A549 and H1299 cells transfected with miR-520a-5p inhibitor compared to inhibitor NC (Fig. 6g).

### MiR-520a-5p inhibitor upregulated CDK4 expression to promote NSCLC cell malignant behaviors

**Fig. 7:**
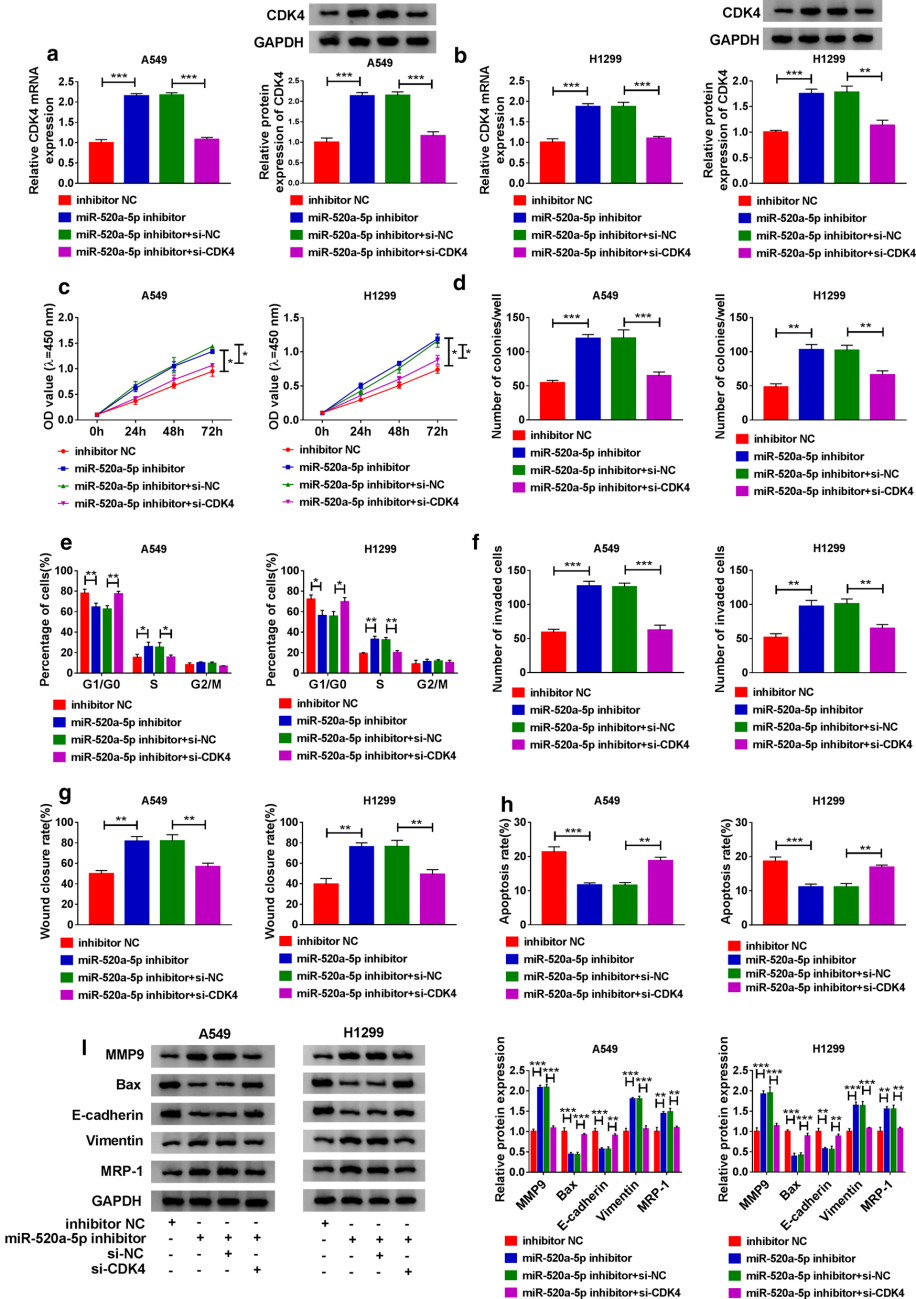
MiR-520a-5p deficiency aggravated DDP resistance and NSCLC development by upregulating CDK4. **a**, **b** The expression of CDK4 at both mRNA and protein levels in A549 and H1299 cells transfected with miR-520a-5p inhibitor, inhibitor NC, miR-520a-5p inhibitor + si-CDK4 or miR-520a-5p inhibitor + si-NC was measured by qPCR and western blot. Then, **c** cell proliferation, **d** colony formation ability, **e** cell cycle progression, **f** cell invasion, **g** cell migration and **h** cell apoptosis in these transfected cells were investigated using CCK-8 assay, colony formation assay, flow cytometry assay, transwell assay, wound healing assay and flow cytometry assay, respectively. **i** The expression of MMP9, Bax, E-cadherin, Vimentin and MRP-1 in these cells was quantified by western blot. *P < 0.05, **P < 0.01, ***P < 0.001

DDP-treated A549 and H1299 cells were transfected with miR-520a-5p inhibitor or miR-520a-5p inhibitor + si-CDK4, with inhibitor NC or miR-520a-5p inhibitor + si-NC as the corresponding control. The expression of CDK4 at both mRNA and protein levels was significantly enhanced in cells transfected with miR-520a-5p inhibitor but largely weakened in cells transfected with miR-520a-5p inhibitor + si-CDK4 (Fig. 7a, b). The abilities of cell proliferation and colony formation were enhanced in A549 and H1299 cells transfected with miR-520a-5p inhibitor but substantially suppressed in cells transfected with miR-520a-5p inhibitor + si-CDK4 (Fig. 7c, d). Besides, miR-520a-5p inhibitor-induced cell cycle progression was blocked by CDK4 knockdown (Fig. 7e). The capacities of cell invasion and migration were induced in A549 and H1299 cells transfected with miR-520a-5p inhibitor but repressed in cells transfected with miR-520a-5p inhibitor + si-CDK4 (Fig. 7f, g). As expected, cell apoptosis was inhibited by miR-520a-5p inhibition but restored by the additional CDK4 knockdown (Fig. 7h). Additionally, the expression of MMP9, Vimentin and MRP-1 was promoted in A549 and H1299 cells transfected with miR-520a-5p inhibitor but repressed in cells transfected with miR-520a-5p inhibitor + si-CDK4, while the expression of Bax and E-cadherin was impaired in cells transfected with miR-520a-5p but recovered in cells transfected with miR-520a-5p inhibitor + si-CDK4 (Fig. 7i). These data suggested that miR-520a-5p deficiency triggered NSCLC cell malignant behaviors by upregulating CDK4.

### Hsa_circ_0014235 activated the expression of CDK4 by targeting miR-520a-5p

**Fig. 8:**
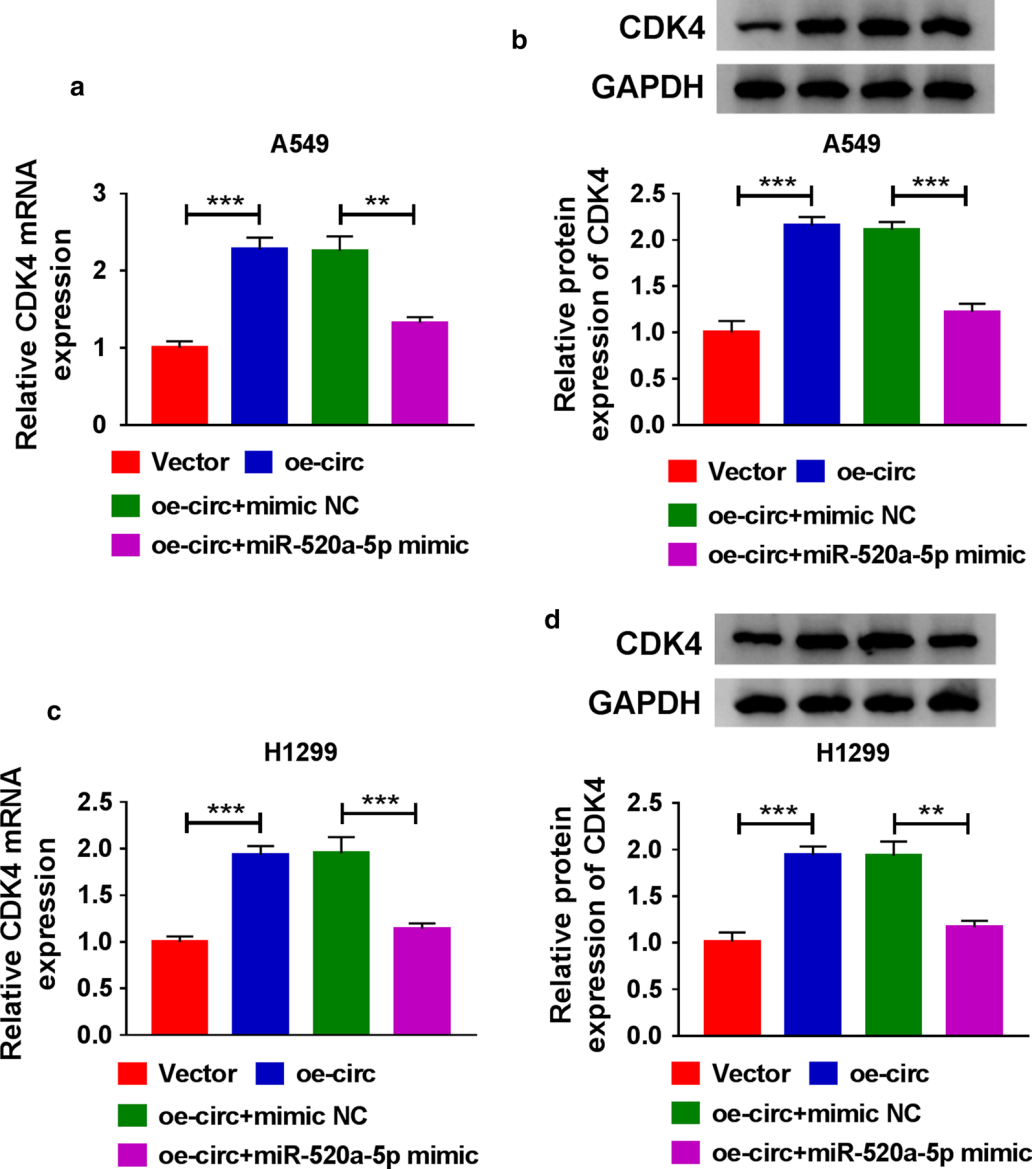
Hsa_circ_0014235 regulated CDK4 expression by targeting miR-520a-5p. **a**, **b** The expression of CDK4 in A549 cells transfected with oe-circ, Vector, oe-circ + miR-520a-5p mimic or oe-circ + mimic NC was detected by qPCR and western blot. **c**, **d** The expression of CDK4 in H1299 cells transfected with oe-circ, Vector, oe-circ + miR-520a-5p mimic or oe-circ + mimic NC was detected by qPCR and western blot. **P < 0.01, ***P < 0.001

To determine whether CDK4 played a part in the hsa_circ_0014235/miR-520a-5p pathway, we detected the expression of CDK4 in A549 and H1299 cells with different transfection. The data showed that the expression of CDK4 at both mRNA and protein levels was highly increased in cells transfected with oe-circ compared to Vector, while CDK4 expression was declined in cells transfected with oe-circ + miR-520a-5p mimic compared to oe-circ + mimic NC (Fig. 8a, d), suggesting that hsa_circ_0014235 positively regulated CDK4 expression by targeting miR-520a-5p.

## Discussion

Over the past few years, numerous therapies have been developed to improve the therapeutic effects and survival rate of NSCLC patients [24]. Even so, developmental drug chemoresistance, frequent recurrence and other poor prognoses are difficult to improve in patients with advanced NSCLC [24]. Hence, early diagnosis is particularly important. Meanwhile, liquid biopsy of tumor can be considered as a non-invasive alternative to tissue biopsy [25], which may be an effective means for molecular diagnosis and management of cancer progression. CircRNAs are shown to be detected in various body fluids, such as saliva and whole blood [26, 27]. Besides, circRNAs are also abundant in exosomes, and exosomal circRNAs are regarded as promising biomarkers for cancer diagnosis [18]. For instance, FLI1 exonic circular RNAs (FECR) could be transferred by serum-derived exosomes in small cell lung cancer patients, and FECR was highly expressed in serum-derived exosomes from small cell lung cancer patients compared with that from healthy donors [28]. FECR was a promising biomarker to predict tumor metastasis in small cell lung cancer [28]. In this study, we found that hsa_circ_0014235 expression was remarkably enhanced in not only NSCLC tumor tissues and cells, but also in NSCLC serum-derived exosomes. Besides, NSCLC serum-derived exosomes harbored high expression of hsa_circ_0014235 could increase DDP chemoresistance and promote NSCLC cell proliferation, migration/invasion and tumor growth *in vivo*.

We strengthened the expression level of hsa_circ_0014235 in NSCLC cells to investigate its function. The results presented that hsa_circ_0014235 overexpression increased DDP resistance, stimulated NSCLC cell proliferation, colony formation, cell cycle progression, invasion and migration but weakened cell apoptosis, suggesting that hsa_circ_0014235 was an oncogene in NSCLC. To determine the mechanism of hsa_circ_0014235 function in NSCLC, the potential miRNAs targeted by hsa_circ_0014235 were identified. As a result, miR-520a-5p was a target of hsa_circ_0014235 and was downregulated in NSCLC tissues and cells. Previous studies showed that miR-520a-5p could enhance the anticarcinogenic effect of capsaicin in chronic myelogenous leukemia [29]. Besides, lncRNA SOX21-AS1 induced cell migration and invasion in triple-negative breast cancer by targeting miR-520a-5p [30]. A recent study reported that miR-520a-5p expression was decreased in NSCLC tissues [31]. Our data displayed that miR-520a-5p enrichment reversed the pro-cancer effects of hsa_circ_0014235 overexpression, hinting that miR-520a-5p might be a tumor suppressor in NSCLC.

MiRNA regulated the expression of mRNAs by binding to their 3’UTR [23]. Based on this, we concluded that miR-520a-5p bound to CDK4 3’UTR and suppressed CDK4 expression by bioinformatics analysis and dual-luciferase reporter assay. CDK4 was reported to be upregulated in lung cancer tissues, and high expression of CDK4 was closely correlated with pathology classification, lymph node metastasis and clinical stage of lung cancer patients [32]. Besides, CDK4 could be targeted by miR-545, and CDK4 overexpression abolished the miR-545-induced inhibition of cell proliferation, thus promoting NSCLC cell proliferation [33]. Similar to these reports, we detected that CDK4 expression was elevated in NSCLC tissues and cells, and rescue experiments in this study showed that CDK4 knockdown abolished the promoting effects of miR-520a-5p inhibition on NSCLC cell proliferation, cell cycle progression, migration and invasion, indicating that miR-520a-5p suppressed NSCLC development by targeting CDK4.

In conclusion, an increase of hsa_circ_0014235 expression was detected in NSCLC serum-derived exosomes, tumor tissues and cells. NSCLC serum-derived exosomes harbored high hsa_circ_0014235 expression promoted DDP resistance, cell proliferation, migration and invasion, as well as tumor growth *in vivo*. Besides, the promoting expression of hsa_circ_0014235 induced NSCLC cell malignant behaviors via mediating the miR-520a-5p/CDK4 pathway. Our study demonstrated the role of hsa_circ_0014235 and provided a potential mechanism for its function, which might be a basic for hsa_circ_0014235 as a biomarker for NSCLC treatment.

## Data Availability

The datasets used and/or analysed during the current study are available from the corresponding author on reasonable request.

## Abbreviations

NSCLC: Non-small cell lung cancer
TEM: Transmission electron microscopy
CDK4: Cyclin-dependent kinase 4
CCK-8: Cell counting kit-8
FECR: FLI1 exonic circular RNAs
